# One Health evaluation of brucellosis control in Kazakhstan

**DOI:** 10.1371/journal.pone.0277118

**Published:** 2022-11-02

**Authors:** Duriya Charypkhan, Simon R. Rüegg

**Affiliations:** 1 Vetsuisse Faculty, Section of Epidemiology, University of Zurich, Zurich, Switzerland; 2 Laboratory of Brucellosis, Kazakh Scientific Research Veterinary Institute, Almaty, Kazakhstan; Washington State University, UNITED STATES

## Abstract

Brucellosis is one of the main livestock disease risks in Kazakhstan. It’s been endemic there since 1930, accounting for over 1300 human cases per annum. The economic loss was 45 million USD in 2015 alone. Since 1952, Kazakhstan has implemented various control strategies with little success. One Health approaches have been suggested to tackle brucellosis, however, there is a lack of evidence for best practices to operationalise One Health in the literature, and methods for implementation are not established. The intention of this study was to introduce the One Health approach during the evaluation phase of the policy cycle. A two-day workshop was organized by the authors to familiarize participants with the evaluation methodology. Twenty-one specialists representing veterinary and public health sector, together with researchers, took part in this study. For two weeks following the workshop, first author conducted individual interviews with workshop participants to obtain individual scorings to assess knowledge integration capacity (One Health-ness). The evaluation results show that there is a lack of knowledge about the perceived damage caused by brucellosis to animal owners and other stakeholders. There is insufficient data available about farmers’ practices, interests and motivations, and also data is missing for important transmission processes such as the amount of unsafe dairy consumption. The absence of such data illustrates the extent of the uncertainty to which decision-makers are exposed despite well-elaborated transmission models and supports the importance of co-producing solutions with participatory methods. The results suggest the need for broader involvement of stakeholders. Outputs of this study could help navigate the initial stages of One Health operationalization.

## Introduction

Brucellosis is a neglected zoonotic disease caused mainly by the Gram-negative bacteria *Brucella abortus*, *B*. *suis* and *B*. *melitensis* [1, 2]. Infections result in abortions in cattle, pigs, goats and sheep respectively. There are instances of host switches, where *B*. *melitensis* was isolated in cattle [3–7]. Due to the reduced reproductive performance of infected animals, brucellosis causes important economic losses [8]. Humans contract brucellosis mainly from direct contact with infected animals (when handling infectious material such as an aborted foetus) or through ingestion of infected animal products (mostly unpasteurized dairy products) [9]. Infections in humans are often chronic and may affect any part of the body, including the cardiovascular, nervous and reproductive systems, culminating in severe ailments. Globally, half a million human cases are reported annually [10, 11].

Throughout the world, various measures are applied to control brucellosis at the population level [11]. The European Commission advises adopting a strategy depending on the prevalence of the disease in large and small ruminants and the accessible resources. These suggestions are of general character and should be adjusted for the particular situation in the affected country or region [12, 13].

The protection of human health relies on eliminating the causative agent from the animal reservoir and preventing zoonotic infections, by pasteurizing milk and thoroughly cooking meat products. Currently, no approved human vaccines exist [2, 14].

In their systematic review on control of brucellosis, Zhang et al. [15] summarize, which measures and strategies were successful in different parts of the world. Several countries obtained OBF status with strategies that took decades and involved meticulous implementation of protocols. Unfortunately, these reports do not comprise successful brucellosis control in low- and middle-income countries (LMICs), apart from Fiji that achieved freedom from *B*. *abortus* in 1996 through the vaccination combined with test-and-slaughter. But even in the case of Fiji, the disease re-emerged due to poor monitoring.

The reported successful strategies are associated with high costs to compensate slaughtered animals, to carry out surveillance in the animal populations, and to perform diagnostic tests. For LMICs these expenses are substantial financial burdens, requiring compromises which may jeopardise successful control [16].

### Situation in Kazakhstan

Kazakhstan, an LMIC and former Soviet Union republic, in Central Asia, gained its independence in 1991. It is the world’s largest landlocked country, neighbouring with Kyrgyzstan, Uzbekistan, Turkmenistan, Russia and China. Agriculture accounts for 5% of the country’s Gross Domestic Product (GDP) producing mainly dairy, meat, wool and leather [17]. Most livestock is owned privately for subsistence farming in the villages [18]. Subsistence farmers use dairy products to cover their family needs and sell some to their village neighbours. About 15% of small ruminants and 25% of cattle are kept by big industrialized farms, both for meat and dairy production [19].

In Kazakhstan, brucellosis in animals has constituted one of the main livestock disease burdens since 1930, therefore in 2015, the Ministry of Agriculture (MoA) requested specific help from the OIE to tackle the disease. In the past decade the incidence in cattle was registered around 0.6 and around 0.4 in small ruminants (sheep and goats) [7]. Human brucellosis has been endemic since the first documented case in 1932 [20]. It presently accounts for more than 1300 notified human cases (7,6 per 100 000 inhabitants) annually, which is among the highest incidences in the world [7, 10].

Up to now, different control strategies have been implemented: from 1952 to 2006, different schemes of vaccination of cattle, sheep and goats were in place, which were replaced by a test-and-slaughter program in 2007. In the latter programme, all animals (registered cattle, sheep and goats) are tested twice a year [19]. Confirmed positive animals are slaughtered and are compensated at market value. In general, up to seventy percent of the compensation is covered by the slaughterhouse or from sales of recoverable meat, and 30% are paid from the government budget [21]. The government compensation is administrated by the local executive office and usually paid after animals have been slaughtered. According to farmers, the price paid by the slaughterhouses never exceeds 50% of the market value, because they define the price per kg meat themselves. The effectiveness of this test-and-slaughter program has stagnated, and the prevalence of brucellosis in ruminants has not decreased in the last four years while substantial economic losses continue. We estimated direct and indirect economic losses due to brucellosis of 45 million USD in 2015 alone [7]. According to the presentations at the regional Food and Agriculture Organisation (FAO) workshop on brucellosis control in Central Asia and Eastern Europe, in 2012, Kazakhstan was the only country compensating owners for both small and large ruminants 100% of the market value. In contrast, for example, the neighbouring country of Kyrgyzstan paid only 75% of the market value [22].

Roth et al. [23] as well as the World Bank Group have recommended integrated One Health (OH) approaches to tackle brucellosis in LMICs [24]. On several occasions, human health professionals in Kazakhstan also underlined the importance of cooperating with the animal health sector [25, 26]. In the recent years, prompted by international organisations, there were attempts to acknowledge the need for One Health and inter-sectoral collaboration. In November 2018, there was a national bridging workshop (NBW) sponsored by the US Defence and Threat Reduction Agency (DTRA). The main objectives were advancing One Health, building sustainable networks and strategical planning of intended changes. The workshop was based on outputs of International Health Regulations (IHR) evaluation and Performance of Veterinary Services (PVS) results for Kazakhstan. Through a series of exercises, participants coming from two sectors (veterinary services and public health officials) had to prioritize the needs in inter-sectoral collaboration [25]. Exercises were based on case examples of some zoonoses, including brucellosis. According to the workshop outputs regarding brucellosis, emergency funding is a technical area that needs improvement. Good collaboration score was given to areas as legislation and regulations, coordination at technical level, communication with stakeholders, joint surveillance, and field investigations. Results also indicate that there is some collaboration noted in the areas of training, communication with media, laboratory and coordination at local and national level. The participants prioritized establishment of joint response system and emergency funding.

In November 2019, FAO and OIE organized a workshop for the establishment of a Central Asian Animal Health Network (CAAHN) with the participation of Kazakhstan, Kyrgyzstan, Uzbekistan, Turkmenistan and Tajikistan. Brucellosis was identified as the initial driver of the network. Recommendations given to the FAO and OIE regional offices were to strengthen the collaboration in the region in a One Health approach [27]. Currently, the public health and veterinary services of Kazakhstan have no joint policies to combat prioritized zoonotic diseases. However, medical doctors and veterinarians share protocols used during brucellosis outbreaks and the epidemiological investigation of human cases [28]. These protocols and the current practices of human and animal health professionals are starting points to develop brucellosis control in Kazakhstan towards an OH approach. The subsequent steps would require transdisciplinary collaboration of multiple stakeholders, overcoming existing barriers between the health sectors and their governing bodies to integrate knowledge [29]. Hitziger et al. [29] suggest methods for knowledge integration at every phase of the policy cycle. However, there is a lack of evidence for best practices to operationalize One Health in the literature, and methods for implementation are not established [30, 31].

For this study, we engaged with the policy cycle in the evaluation phase and assessed the knowledge integration capacity of the current national brucellosis control strategy through evaluation. We employed a systems approach to evaluation following the procedures proposed by the EU COST Action “Network for Evaluation of One Health (NEOH)” [32]. One Health requires multi-stakeholder knowledge to be synthesised into a coherent narrative, which can only be achieved through translation of diverse individual narratives using systems thinking. The knowledge generated in the evaluation informs the development of the policy for brucellosis control towards a more systemic and inclusive OH approach.

## Materials and methods

### Ethics statement

The present project doesn’t fall within the scope of the Human Research Act in the Swiss or Kazakh legislation, because we did not process data related to personal health conditions nor did we include any sampling, experimentation with humans or engage in interventions. This was confirmed through a checklist provided by the ethics commission of the canton of Zurich [33]. The present study can therefore be regarded analogous to a marketing investigation or journalistic work. To adhere to good scientific practice, verbal consent was sought from all participants prior to the workshop and interviews. All had been informed about the aims of the study and methods upon the arrival to the first meeting. All data was treated confidentially.

### Study design

The NEOH evaluation framework regards OH initiatives as complex adaptive systems within the larger context in which they operate. It combines descriptive and qualitative assessments in a mixed methods approach, and was validated in a set of case studies [34]. For the current project, basic knowledge was acquired with a literature search (S1 File), and followed by a participatory mapping of the system to develop a collective understanding of the (brucellosis control) system at stake. In our study, we focussed on the assessment of knowledge integration capacity and used the corresponding NEOH tool to assess One Health-ness, and a refined, more generic tool for evaluating knowledge integration capacity in multi-stakeholder governance (EVOLvINC). NEOH tool allows to produce a One Health ratio and One Health index, the metrics, that can be compared across One Health initiatives. EVOLvINC assesses the integration of knowledge through the governance processes in multi-stakeholder science-policy initiatives [32, 35].

### Workshop and participatory mapping

In November 2018, the two authors of this manuscript proposed a workshop to map brucellosis control and evaluate the current brucellosis control policy to the veterinary research institute in Kazakhstan (KSRVI). First author is a national of Kazakhstan and has previously been involved in research on brucellosis at the KSRVI. Thus, the institute acted as a mediatory agent, and in turn engaged the Chief Veterinary Officer of Kazakhstan, the MoA and representatives of local division of the Ministry of Health (MoH). The respective institutions were contacted via email to explain the study aims and request for a collaboration. Each institution designated relevant participants. In January 2019, a two-day workshop was conducted in Russian at the KSRVI. The aim of the workshop was to present the OH approach including system thinking, introduce the NEOH evaluation tool, and initiate discussions about the possibilities to improve brucellosis control strategies. The verbal consent was obtained from each participant prior to the workshop. The program consisted of presentations introducing the NEOH framework, some case examples [32, 34, 36] and a series of mapping exercises to develop a common understanding of brucellosis control in Kazakhstan. Twenty-one specialists and professionals appointed by the MoA and MoH, as well as academics, attended the workshop. The MoA was primarily represented by the Committee of Veterinary Control and Supervision (CoVCS): attendants were veterinary inspectors at regional levels, specialists from the Republican anti-epizootic squad and the veterinary diagnostic laboratories, as well as private veterinarians. Attendants from the MoH were medical practitioners dealing with human brucellosis at the regional level. Academic researchers involved in studies on brucellosis were also present. At the time of the workshop, all participants were involved in administering and implementing veterinary control measures, human and animal diagnostics, and post-outbreak management. There was also one specialist involved in the formulation of policy and veterinary regulations.

As an introduction, participants were briefed using an activation method of Tom Wujec (https://www.drawtoast.com/). They were then assigned to four groups, balanced for sectors and disciplines, and were instructed to develop their common perspective of brucellosis control in Kazakhstan as concept maps on flip charts [37]. Besides representing the collective understanding of brucellosis control in Kazakhstan, the maps also acted as a boundary object for the conversation. These maps were successively merged in further brainstorming and mapping exercises. Throughout the whole process, the participants engaged in open conversations and discussions about issues that may obstruct the results of the current control strategy. The main facilitator (DC) led the workshop and took notes along with the process, the second facilitator, co-author of the NEOH framework and the EVOLvINC tool, but non-Russian speaker, observed the group dynamics and the timeline, and acted as a reference for questions.

### Interviews

The aim of the interviews was to score six different aspects of knowledge integration that are essential for One Health initiatives. The interviews were conducted face-to-face during the two weeks following the workshop. The verbal consent from each participant was obtained prior to the interview. They were guided by the questions from the NEOH “One Health-ness” assessment tool (S2 File) [34]. The tool consists of some open-ended questions and questions that are scored on a four-level Likert scale. The original questions were translated to Russian by the first author (DC) and the translation was validated through back-translation to English using an independent, commercial translation service. The interviews were conducted in Russian and lasted approximately two hours each. Notes were taken in the presence of the participants and the questionnaire was completed together with the interviewee. Subsequently, the questions were scored and validated in the presence of the interviewees. The conversations were audio-recorded for later reference, but not transcribed verbatim nor translated. The interview responses were entered into an excel file the same day.

Because of varying levels of fluency in Russian, and technical vocabulary associated with systems thinking, the interviewer was asked in some cases to translate some words or sentences into Kazakh for clarification. Sometimes, the interviewer rephrased questions into easier sentences to help with the main concepts. In earlier work with the NEOH framework it became clear that difficulties arise with the acquisition of systems thinking [34]. Therefore, to identify the predominant hurdles to transit towards a systemic appreciation of complex problems, interviewees were also asked the following two questions (S3 File):

1. Is there any specific obstacle you encountered during introduction to the systems approach?
2. In retrospection, how would you have found it easiest to learn systems theory?

The interview data was anonymized, de-identified and triangulated with legal documents, reports and legislation for validation. De-identification and anonymization were done for data privacy, to warrant that conclusions drawn from the process could not be traced back to individual participants. Triangulation was primarily done to support individual statements with independent data and enhance its systemic validity. Questions that deal with perceptions were not triangulated.

The text and audio files are stored on the department server, to which only department employees have access. The first author keeps a personal copy of the data.

### System mapping

**Fig 1:**
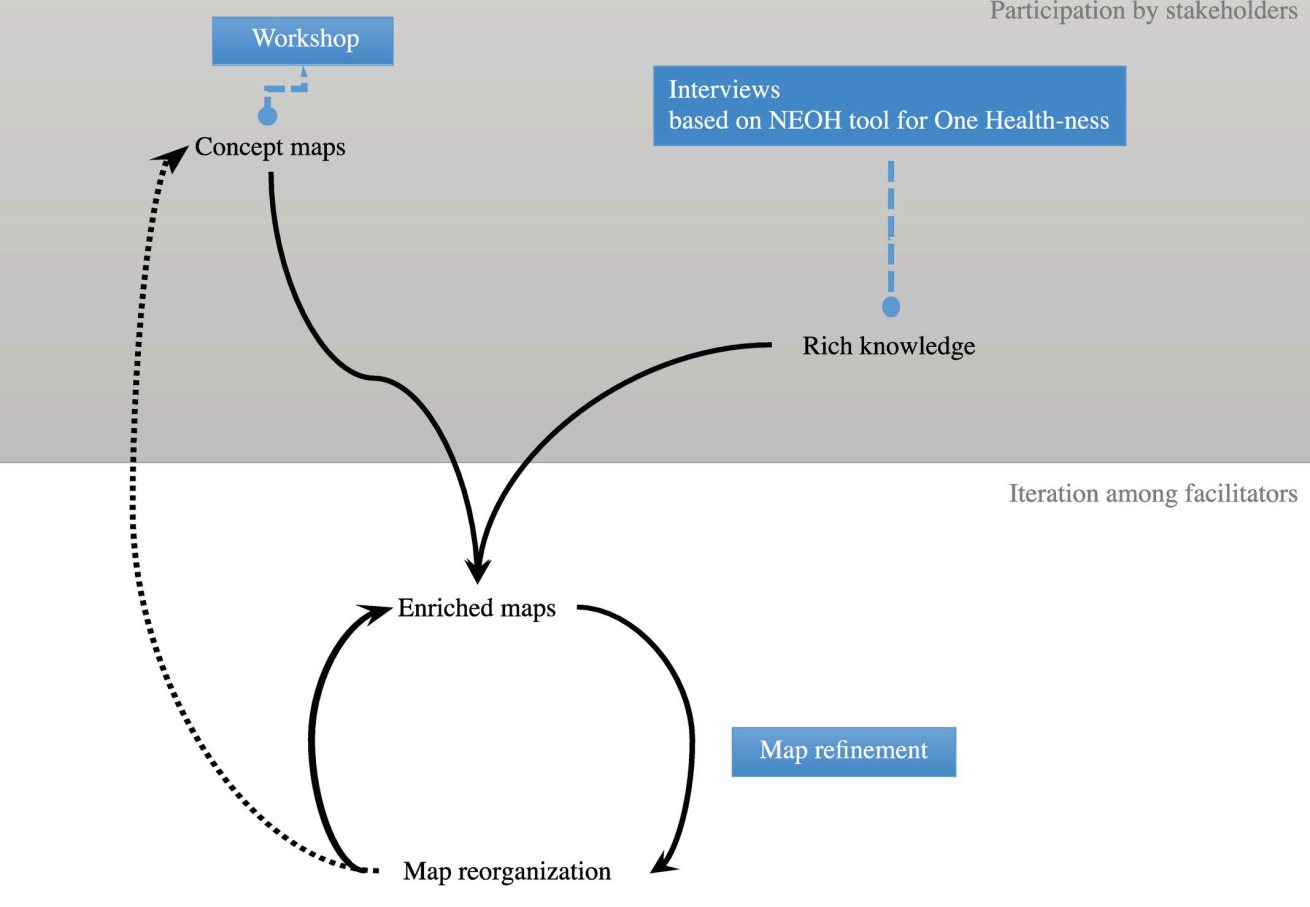
Schematic presentation of the iterative process of the map refinement.

Physical copies of the maps produced during the workshop were digitalised using the software CMap (https://cmap.ihmc.us/cmaptools/). In a process based on grounded theory, all elements and links used by participants were entered into a draft, juxtaposed and merged. The missing links and elements were added using information extracted during the interviews. The interviews provided knowledge about the broader context of the specific question and allowed to enrich the system maps with elements and behaviours that were mentioned by participants and were not voiced during the workshop (Fig 1). In an iterative process between the authors, the maps were refined and restructured.

### Assessing knowledge integration capacity

The NEOH tool (S4 File) to assess One Health-ness was used to generate the scores for “thinking”, “planning”, “working”, “learning”, “sharing” and “systemic organisation” [38]. For each question, a score between 0 and 1 (weak to strong expression of the indicator) was attributed. Nineteen participants of the workshop went through interviews (2 withdrew). Participants coming from the other districts were scheduled in the days after the workshop to abridge their travel time. One participant withdrew due to personal travel arrangements. Upon follow-up, the participant also declined an interview by phone or conducting it at another time. The second non-participant did not feel comfortable about attending the interview without giving a reason.

The scores given by interviewees were aggregated to a median score for each question. Then the OH index and ratio were computed using the NEOH tool [38], i.e. the median score of all questions associated to an aspect is plotted as a corresponding spoke in the spider diagram. The resulting hexagonal surface represents the degree of integration and numerically is interpreted as the One Health Index. The OH ratio is the relation of the surface covered on the top left (OH operations) of the diagonal to the one on the lower right (OH infrastructure). The EVOLvINC tool (S5 File) was used to aggregate the relevant questions and assess knowledge integration capacities in the multi-stakeholder governance cycle [35].

## Results

### System mapping

Participants produced 12 maps with varying degrees of structure, interconnectedness and complexity. During the mapping process, each group discussed their perspectives until they reached consensus about the elements they would include in their map. The most common elements of the maps were the genus of the infectious agent (“*Brucella*”) or the term “brucellosis” for the disease. Further frequent elements were “veterinarian”, “sanitary measures”, “human doctor”, “compensation” and “diagnostics”. There was a consensus among participants that the current control program mainly addresses the infectious agent through bi-annual screenings. Elements on the maps differed between groups according to their composition. For e.g. a group including a field veterinarian and veterinary inspector primarily used terminology from veterinary regulations for brucellosis (quarantine, diagnostics, movement control, etc.). The group with a researcher who had worked on brucellosis concentrated more on diseases indicators. Participants struggled to depict the system as a causal loop diagram to illustrate the dynamics of the system.

**Fig 2:**
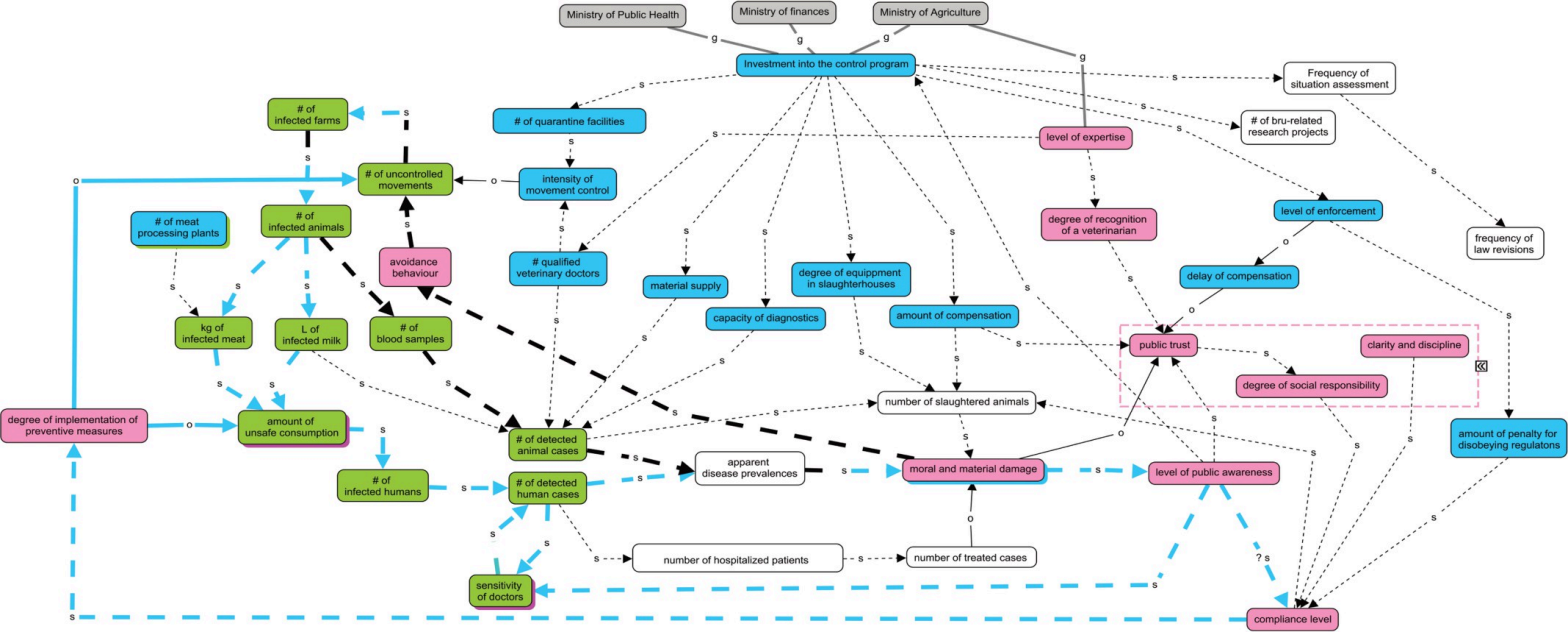
Map of the Kazakh system for brucellosis control. The boxes represent governing systems (grey), components affiliated with environmental domain (green), the economic domain (blue) and the social domain (pink). Shadows around elements indicate their affiliation to a secondary domain. Causal relationships are represented with black arrows where “s” stands for the change in the same direction (dashed line) and “o” (solid line) for the opposite direction. Grey lines with letter “g” represent governance links. Loops: a balancing loop (blue) reduces the “amount of unsafe consumption” and “uncontrolled animal movements” via “public awareness” and “compliance”. A reinforcing loop (bold black) raises the “number of infected animals” via “avoidance behaviour” and “uncontrolled movements”. If limited resources need to be allocated, the relative strength of these loops will determine the disease prevalence.

The workshop maps were merged by the authors and enriched with the interview material (Fig 2). During the iterative refinement, three main domains emerged referring to ecological, economic, and social aspects. The ecological domain (green boxes) represents the disease transmission, the economic domain (blue boxes) consists of elements that are (primarily) funded by the governmental budget, and in the social/behavioural domain (pink boxes), participants referred to “mentality” issues.

In the map in Fig 2, this overarching term of mentality contains the elements “public trust”, “degree of social responsibility” and “clarity and discipline” outlined by a pink broken line. There was uncertainty about the quality of the link between “level of public awareness” and “compliance level”, because it was not clear whether a raised “level of public awareness” would potentially lead to decreased or raised “compliance level”. Another complex element is “avoidance behaviours”, which include but are not limited to home slaughters without prior veterinary inspection, giving infected animals as gifts for social gatherings or celebrations (such as weddings, birthdays, school graduation etc.), and selling animals independently without any official certification from veterinary departments. Giving infected animals contributes to uncontrolled movement and the potential spread of the disease. The value of an animal given as a present is perceived higher than the revenue generated when it is slaughtered due to confirmed infection in the herd.

There are interesting links between the domains. While investment in various executive capacities clearly affects the case detection and the monitoring of the ecological domain, the investment in “expertise” and “enforcement” additionally affects “public trust” and “compliance level”. In return, the social domain may affect investment in a reinforcing loop and influence the “number of uncontrolled movements” through the “degree of implementation of preventive measures” or “avoidance behaviour”. The ecological domain is linked to the social domain only through the “moral and material damage” caused by the disease. Three feedback loops are noteworthy: 1) The loop “human diagnostic sensitivity” describes a reinforcing feedback as “moral and material damage” caused by brucellosis causes a change in the “level of the public awareness” in the same way, which in turn will affect the “sensitivity of doctors” and the “number of detected human cases” representing a component of the “moral and material damage”. 2) Additionally, a balancing and a reinforcing loop converge in “moral and material damage”. The balancing loop initiates with the “level of public awareness” changing as a result of a “moral and material damage” producing a balancing feedback via detected “number of detected human cases”, and all elements change in the same direction except for the “degree of implementation of preventative measures” influencing the “amount of unsafe consumption” in the opposite direction. This suggests that there is an equilibrium between the “public awareness” of the damage caused by brucellosis in human health and the cost of implementing preventive measures. The reinforcing loop develops over the “avoidance behaviour” from “moral and material damage” raising the “number of detected animal cases” etc.

### Acquisition of system thinking

The participants of the workshop outlined that if examples of other system maps were introduced before the exercises, it could have been easier to navigate through the mapping process and create more “sensible” maps. Four respondents found mapping sessions to be boring and proposed to use different exercises to mix things up. The major obstacle is believed to be the terminology. The questions regarding acquisition of systems thinking were not used in previous case studies, hence are not validated. Care was taken to use the correct technical terms for the Russian translation (and translated back for validation), but only two participants had been exposed to systems thinking prior to the workshop. Some interview questions were difficult to understand ad hoc so all participants wished a “simplification” of the terminology. Most questions were very specific and required an understanding of the knowledge integration processes. This stands in contrast to the conversations during the workshop, which were casual in common terms in both Kazakh and Russian and did not pose any difficulty to the participants. Two of the interview respondents shared in confidence, that they struggled with the terms but didn’t acknowledge it during the workshop indicating that group dynamics may have obscured this aspect. Respondents suggested sending out study materials beforehand to acquire and process new concepts. One respondent said that they as actors and agents implementing the control strategy are not sensitised to systems thinking: The aims of the control program are prescribed and require them to implement the protocol. They are conditioned to react immediately to the event–outbreak of brucellosis. All participants agreed that the workshop was helpful to grasp the main concepts of systems thinking allowing them to answer the questionnaire. However, it required huge concentration efforts (This was confirmed by four cases when respondents got tired throughout the interview and asked for a break). They suggested conducting a workshop that lasts longer and offers broad learning opportunities.

### NEOH One Health-ness tool

**Fig 3:**
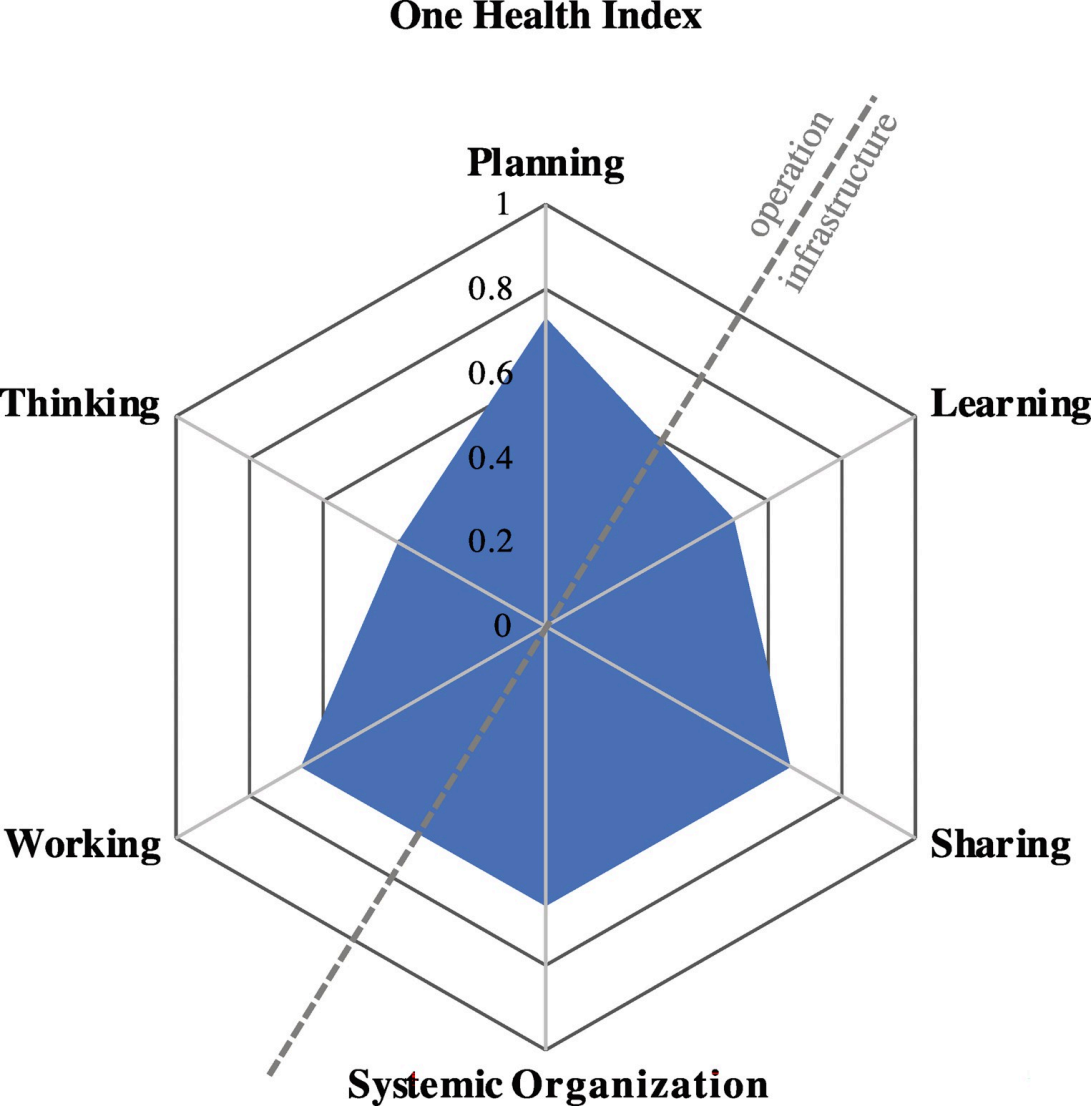
One Health index and ratio according to Rüegg et al. **[38].** Each spoke of the spider diagram represents the median score of an aspect, i.e., thinking, planning, working (operations) and learning, sharing, systemic organisation (infrastructure). The surface of the enclosed hexagram corresponds to the One Health index, the fraction of the surface at the top left (operations) to the bottom right surface (infrastructure) is the One Health ratio.

The minimal dataset and detailed scoring of the evaluation questions are provided as Supporting Information (S1 Table and S6 File). The One Health Index was 0.36 and the OH ratio 0.87. They are graphically represented in Fig 3.

The score for “thinking” was 0.4. Questions scored below 0.5 referred to system features and targets, perspectives and factors relating to the theory of change. Questions referring to initiative-to-environment match and integrated health approaches both scored 0.8. Sustainability and socio-ecological considerations scored 0.5.

The “planning” score was 0.73. It is due to high scores (0.9) attributed to stakeholder and actor engagement, frequent self-assessments, and plan revisions. The allocated budget and time for each objective of the control program were believed to be adequate.

The “working” aspect scored 0.7, and reflects the strongly hierarchical structures, the established chain of command, and clear objectives and responsibilities for each actor. The respondents, also actors in the system, were aware of the tasks assigned to them, and familiar with the regulations and protocols to implement in every scenario. Teams at all administrative levels are highly organised. There is an internal coordination mechanism with a clear chain of command for all prescribed activities. All team members are aware of their obligations, and responsibilities are clearly delineated. The highest score (1.0) was attributed to cultural and social balance indicating that the power distribution across genders, social classes, religions and disciplines is perceived as balanced. In their institutions and across disciplines there are representatives of Kazakhs, Uighurs, Russians, Dungan, Turkish, Uzbeks etc. comprising Muslims, atheists and Christians. Out of 19 people interviewed, there were 5 female respondents, representatives of different social classes, of different cultures and religions. It is noteworthy that all respondents were representatives of the decision-making organisms. Other stakeholders such as farmers or slaughterhouse managers who were not involved may have a very different perception of the power balance.

The power distribution across sectors was scored 0.66, reflecting an advantage in favour of veterinary health professionals, as they are the main implementers of the control program. Leadership is believed to be open-minded to creative input, and internal hierarchies are adequately flexible in decision making with regards to changing circumstances and adapting to tasks. The lowest score (0.4) was given to the flexibility of the control strategy to changes in project design and timeline. It is only able of long-term adjustments. For example, since 2014, the MoA is attempting to revise the current strategy, with the help of OIE experts. The draft recommendations are discussed with state veterinarians at all administrative levels, and with stakeholders such as academia, researchers, and the farmers’ association.

The “systemic organisation” score was 0.66, with the lowest score (0.5) given to social and leadership structures and skills that address hierarchies and management style. A high score (0.9) was given to team structures.

The score for “sharing” was 0.66. Data sharing is easy between sectors (MoH and MoA) and between levels across sectors and across organisations. MoA has divisions of CoVCS in each region and district throughout the country, as well as separate institutions that it oversees (republican veterinary laboratories, national veterinary reference centre, republican anti-epizootic squad, veterinary research institute). MoH also has several divisions, departments and committees throughout the country. The information and data exchange occur within the divisions on various levels and across disciplines, vertically and horizontally. Interviewees’ responses on this subject were concordant with existing official protocols and regulations regarding the information and data exchange. Respondents outlined that the information is reliable, however they criticised that human errors occur occasionally, because of lack of attention.

The “learning” aspect scored 0.51. The policy supports team and organisational learning and highly encourages adaptive and generative learning. Team members attend several continuing education trainings, special seminars organised by the programme, and continuously develop their general skills and knowledge on operational tasks. But on an individual level (scored 0.4), there is a lack of motivation. This may partially be because field veterinarians, according to respondents, earn around 150 USD per month, which is not perceived as satisfactory and not adequate for the workload.

### EVOLvINC tool

#### Agenda setting and formulation phase (thinking and planning)

**Fig 4:**
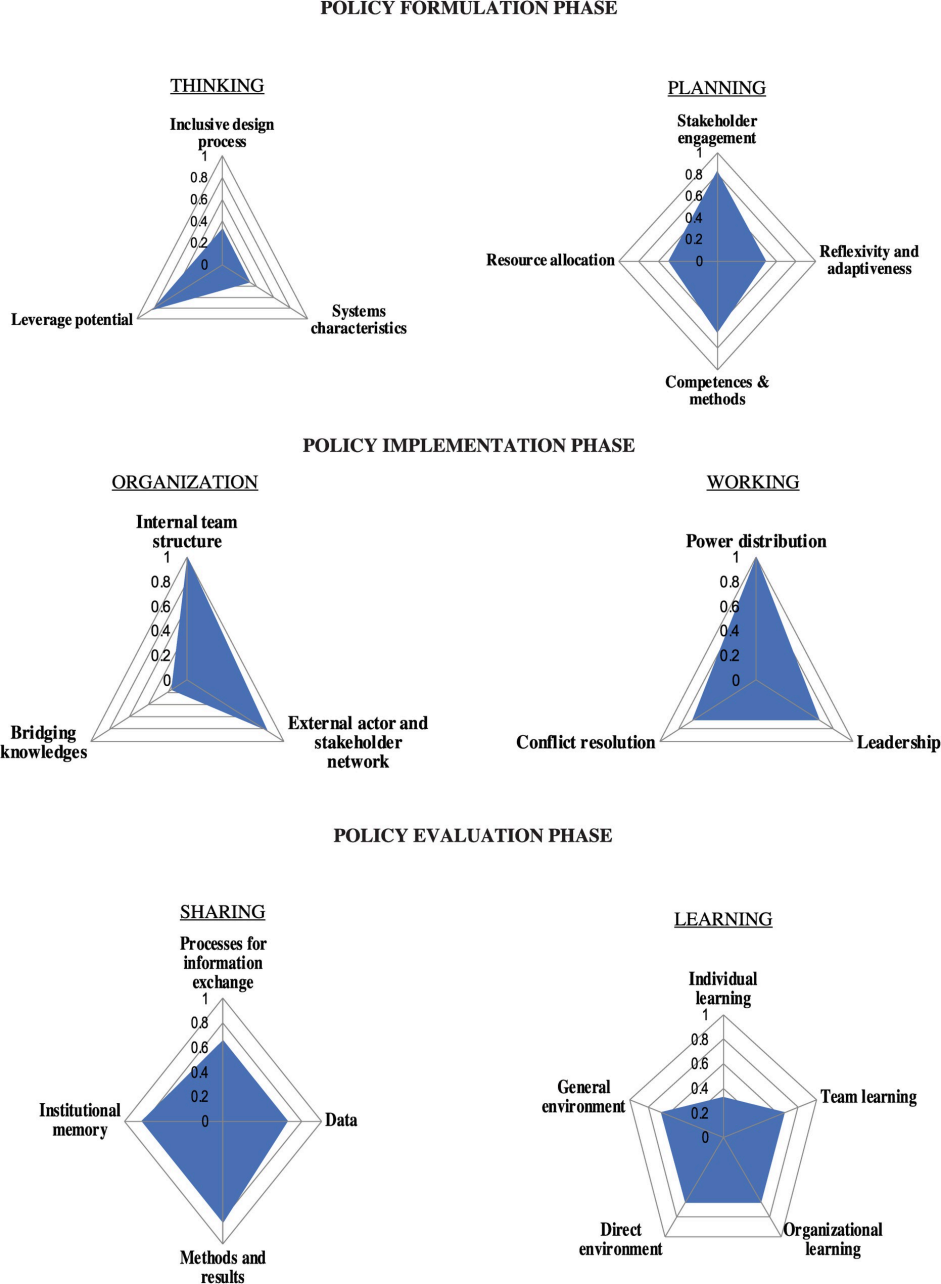
Evaluation results obtained with the EVOLvINC tool for the aspects contributing to the knowledge integration capacity during the formulation phase, implementation phase and evaluation phase of the policy cycle. Scores are medians and range from zero (not supportive for knowledge integration) to one (highly supportive for knowledge integration). The figure indicates that there is potential to improve the systems approach for the “formulation” phase. “Implementation” appears to benefit primarily from “bridging knowledges”. “Evaluation” seems well developed, which was confirmed by the engagement of participants in the project. Data was collected during personal interviews in 2019.

The inclusive design process and the consideration of system characteristics scored 0.33 (Fig 4). The problem of brucellosis is well translated into scientific and developmental questions explaining the score of 0.83 for the leverage potential. The scientific questions in Kazakhstan are addressed by research institutes and universities by mandate of the MoA or through research grants. Most projects concern the biology of *Brucella* and the aetiology of the disease, epidemiological research, as well as the development of new diagnostic tools and vaccines. The developmental questions are addressed by CoVCS.

The policy addresses brucellosis outbreaks as singular events, and little attention is given to patterns or underlying systemic structures. Almost no consideration is given to feedback loops, and the policy itself is not conceptualised as an evolving complex system.

#### Policy implementation phase (organisation and working)

Arising conflicts and tensions are resolved on site through mediations and impositions. The MoA and the MoH prescribe procedural protocols when new cases of brucellosis are registered. The lowest score was obtained for bridging knowledge, implying that methods and process to integrate knowledge from stakeholders, actors and team members are restricted to facilitated dialogues and centralised integration by the project management.

#### Policy evaluation phase (sharing and learning)

The institutional memory of the initiative relies only on document archives and publications. General information is communicated to each team member on a regular basis, and there are several workshops, trainings and meetings aimed at procedural and technical improvements. Hence, basic and adaptive learning is in place. The direct environment (actors, stakeholders, institutions involved in the program) is open to promote learning processes. It is important to note that there is no central data storage such as a centralized server (database), but information is shared via forms, and there is a clear reporting protocol along the hierarchy.

## Discussion

Unintentionally, the three domains of ecological, economic and social elements emerged during the map refinement. They resonate the findings in earlier workshops on One Health approaches that these imply the three pillars of sustainable development [36], and a certain bias by the authors cannot be ruled out. In the current case, “moral and material damage” caused by brucellosis appears to play a pivotal role between the three domains. Apart from apparent direct economic losses for animal owners, there are several effects of brucellosis in humans, accounting for direct, indirect and intangible costs associated with physical pain and emotional suffering [31]. It is noteworthy that the balancing and reinforcing loop develop over the diagnosis of animal cases or human cases, respectively. This implies that investment in diagnostic capacity in the animal sector potentially increases the risk of human brucellosis if it is not kept in balance by the capacities in the human health sector. Consequently, more identified cases in animals (to be slaughtered) increases “avoidance behaviour”, e.g. owners give infected animals as gifts instead, contributing to uncontrolled movement and the spread of the infection within the animal and subsequently the human population. This pattern was also observed in Bedouin communities: animal owners incorporated illegal trade routes due to late and insufficient compensations to escape the culling procedures [39]. In contrast, more identified human cases reinforce the sensitivity of doctors and awareness of people, which is expected to have a positive effect on compliance. Both loops contain an element which describes a complex of behaviours by stakeholders requiring more detailed enquiry: a) it is unclear if higher awareness about brucellosis will increase the level of compliance or if it leads to more avoidance behaviour, and it may also be different in different stakeholder groups b) it is also not clear what the drivers of avoidance behaviour are. In conclusion, this emphasises that the participants have little knowledge about how stakeholders perceive “moral and material damage”. A potentially similar situation was observed in Negev, where more than 3400 animals were culled for brucellosis control. The owners were compensated with half of the market value for the infected animal. Authorities assumed that with the compensation for the culled animals, farmers could rear new herds. However, Bedouin owners felt discouraged stating that the value of an animal goes beyond its full market value, and was associated with emotional attachment to the herds [39].

A further important node in the map with a high in-degree is “public trust”. It is inversely related to the “moral and material damage” and influenced by the “amount of compensation” as well as the “delay of compensation”. The first refers to payments that are often less than the market value for healthy animals, because the positive brucellosis test is considered as a value reduction on the market. The second results from a long bureaucratic process to have access to the compensation by the government. In 2016, for example, an online news platform reported that about 69 owners of the West Kazakhstan region were waiting for the governmental compensation amounting to 50’295 USD [40]. Only after a letter from the district prosecutor’s office was sent to the local executive body, the compensation was implemented. The system map suggests that reduced “public trust” due to a “delay of compensation” shifts stakeholders’ behaviours from the balancing loop through “compliance” towards the reinforcing loop with “avoidance behaviour” [41]. A lack of public trust is also attested by the NGO Transparency International, which reports a corruption perception index for Kazakhstan of 31%. This index accounts for bribery, diversion of public funds, and excessive bureaucratic burden that can increase opportunities for corruption, among other aspects [42]. The observation that elements contributing to this negative perception appear on a system dynamic map without explicitly probing for corruption and that they are noted as positive features (“trust”) suggests that systems approach to health may contribute to a path towards more transparency. This concurs with a recent study on opportunities for brucellosis control in Mexico. The authors underline the importance of a participatory control strategy to understand farmer’s practices, interests and motivations [43]. Giving stakeholders a chance to voice their opinions and feelings is instrumental to increase trust and cooperation [44].

The transmission pathways represented in the ecological domain contain elements that are not considered in existing infection models [45–47]. Moreover, many data that would be required for such models are missing, e.g. “amount of unsafe consumption”, the key in the transmission of zoonotic diseases. The absence of such data illustrates the extent of the uncertainty to which decision-makers are exposed despite well-elaborated transmission models and emphasises once again the importance of co-producing solutions with participatory methods.

In many ways, the current policy to control brucellosis in Kazakhstan performs well in the eyes of the consulted stakeholders. The main actor of the brucellosis control is the MoA, with different supervised subdivisions responsible for the implementation of the programme [28]. The work of the whole team is highly structured and each team in the organisation and team member has roles and duties clearly described in the documentation related to the control program [48]. The collaboration of the MoH and MoA is considered to be well developed to handle brucellosis outbreaks and fit to be used to further improve knowledge integration.

A highlight is that the sharing of data is considered easy and not bureaucratic as it relies on a legislative framework [49]; the score is at 0.66 due to questions on sharing mechanisms, processes and data quality. This is despite a lack of digital tools and infrastructure.

Also, the “working” score (0.7) indicates that the collaboration across sectors works well. However, the system map suggests some room for improvement with more participation by farmers and other stakeholders concerned by brucellosis. The “planning” and “systemic organization” scores point towards adequate budgetary resources for the management of brucellosis in Kazakhstan, although it neglects the contribution by slaughterhouses, which is considered insufficient. The current brucellosis control program is primarily funded by the government [50]. Veterinary activities are covered by the budget program №249 which regulates creation of conditions for livestock production (through disease prevention and management, disease and food safety surveillance and diagnostics etc.). All expenses associated with procurement of the diagnostic tools and serological tests on serum samples are covered by this program. Culled animals are compensated to the animal owners [28]. The budget for 30% of the compensation payments is provided by local executive bodies at each administrative level. The remaining 70% should come from the slaughterhouses, however they seem not to comply to this requirement.

From the perspective of developing a holistic initiative with balanced operational aspects and supporting infrastructure, “thinking” and “learning” appear to be the biggest challenges. The EVOLvINC tool further highlights a need for more knowledge bridging. The “thinking” score indicates that the policy is not conceptualised as an evolving system and takes no reference to possible feedback loops or the dimensions beyond the presence of disease and associated costs. The focus is clearly on human and animal health as an ecological aspect of OH, with economic considerations, but little thought on social sustainability or wider environmental aspects. For comparison, Malta (“thinking” score 0.8) eliminated brucellosis through a broad integrated health approach that had evolved over decades and was modified through failed attempts [51]. The failed attempts were associated to the unwillingness of the general public to stop consuming unpasteurized dairy products and uncontrolled animal movements. Only after the creation of the intersectoral outbreak committee, strong enforcement on farmers to notify animal movements, thorough awareness campaigns, and the repeated communication of the consequences of consuming unpasteurized cheeselets to the public, it became possible to control the disease. Ultimately, the ban on the sale of fresh cheeselets, their confiscation, and the destruction of all identified infected animals allowed the elimination of human brucellosis in Malta. The success required strong cooperation not only of health inspectorates, both in humans and animals but also with the Department of Consumer Affairs. The Maltese success highlights the importance of applying the right measures at the right time, relying on functional infrastructure. The Kazakh case evaluation demonstrates that the infrastructure between the MoA and the MoH is established and appropriate for further collaborative action. However, the variability of husbandry practices in Kazakhstan and presence of both bovine and small ruminants brucellosis combined with a vast territory add significant challenges [19].

The majority of participants of this study were the representatives of the decision-making organisms. They were appointed to take part in this study because of their expertise on brucellosis. All the participants were directly involved in the brucellosis control program at some stage of their career. For example, the researchers are conducting brucellosis research for the past 25–45 years. They also represent a scientific team that advises the Ministry of Agriculture on brucellosis related questions. The representatives of the committee of veterinary control and supervision have at least 5 years of experience in the field of the legislative framework regarding brucellosis. The public health representatives were involved in human brucellosis control in the region and had varying levels of expertise (from 3 to 30 years of experience). Although, the veterinarians that were present in this study implement the control program on site and have the knowledge about the avoidance behavior of the farmers they might not have the knowledge of their explicit motivations. To some extent, perspectives and motives of all participants represent those of their respective organisations. Therefore, the results of this evaluation should be interpreted with caution.

Despite triangulation with data from official documents, dishonesty and/or lack of knowledge cannot be entirely excluded. The results suggest that some stakeholder perspectives are missing. The method requires trusted relationships. Particularly sensitive subjects may have been omitted if an external evaluator had conducted the assessment. On the other hand, the fact that the main facilitator was “insider” could have introduced bias to the study too.

As mentioned earlier, One Health requires multi-stakeholder knowledge to be synthesised into a coherent narrative, which can only be achieved through translation of diverse individual narratives using systems thinking. The initial workshop allowed to explain the concepts and brief the participants for a broader reflection. Participants struggled with systems thinking and the associated terminology during the mapping exercises and the interviews even if the general questionnaire was produced in an iterative, participatory process and validated during NEOH case-studies in other countries [34]. It confirms previous experiences that system thinking requires time and perseverance.

In conclusion, there is a clear need for a better knowledge about further stakeholder perspectives, such as farmers, slaughterhouse managers, physicians and brucellosis patients. Further work should be done with other stakeholders to complement the findings. Co-producing an adaptive strategy for brucellosis control would require a careful assessment of the learning opportunities at all scales and for all stakeholders, and an infrastructure for sharing data, ensuring its accuracy, warranting its privacy and its long-term storage.

## Supporting information

S1 File Literature search protocol. (PDF)

S2 File Full questionnaire. (PDF)

S3 File Interview guide. (PDF)

S4 File. NEOH tool (XLSX)

S5 File Evolvinc tool. (XLSX)

S6 File Detailed scoring of the evaluation questions (comparison between NEOH and Evolvinc tools). (PDF)

S1 Table Dataset. Excel file with the interview scores.
(XLSX)
