# Supplementary material for: One Health evaluation of brucellosis control in Kazakhstan
Source: PLoS One. 2022 Nov 2;17(11):e0277118. doi: 10.1371/journal.pone.0277118 (PMC9629608; doi:10.1371/journal.pone.0277118)
Supplement: S1 File — (PDF) [file pone.0277118.s001.pdf]

S1 File. Literature search

The non-systematic literature review focused on the articles relevant for our study using the search terms “*Brucell* AND Kazakh* AND (One Health OR integrated OR transdisciplinary) AND control*” in Scopus and PubMed.

Database CyberLeninka (https://cyberleninka.ru) was used for manuscripts written in Russian language with the Syntax: *“Бруцеллез в Казахстане”* @keywords *единое здравоохранение*.

In addition, we analysed legal acts, resolutions of the government, orders of the ministries, directions, regulations and protocols. They were retrieved from Legal information system of Regulatory Legal Acts of the Republic of Kazakhstan (http://adilet.zan.kz/) using the terms “brucellosis”, “regulations on compensation”, “regulations on slaughter”, “regulations on diagnostic tests” in Russian (Table 1).

Table 1. Corresponding terms in Russian used in the search at http://adilet.zan.kz/

brucellosis	Бруцеллез
regulations on compensation	Правила по выплате компенсации
regulations on slaughter	Правила убоя
regulations on diagnostic tests	Правила по диагностическим тестам

Lay publications and public media were screened to gather additional information on the matter of brucellosis control and perceptions of the public. Materials that were relevant to the topic were included. It was conducted using search engines as Google Search (https://www.google.com) and Yandex (https://yandex.ru).
