# Supplementary material for: One Health evaluation of brucellosis control in Kazakhstan
Source: PLoS One. 2022 Nov 2;17(11):e0277118. doi: 10.1371/journal.pone.0277118 (PMC9629608; doi:10.1371/journal.pone.0277118)
Supplement: S2 File — (PDF) [file pone.0277118.s002.pdf]

Questionnaire for the process evaluation of transdisciplinary or intersectoral initiatives

1. Personal information and role of the respondent

1.1 Your name and title		1.2 Date, location
1.3 To which institution are you affiliated?	1.4 What is your current position?	1.5 Please state your email and phone contacts?
1.6 What is the title or name of the project or initiative that you are working in?		
1.7 Where does this initiative take place (country, district, city)?		
1.8 What is your role in this initiative?		
1.9 What would you consider your key contribution to this initiative?		

2. Definition of context and initiative

2.1 What is the problem / information gap that your initiative addresses / tries to fill? This question refers to your conceptualization of the problem or information gap, which the initiative aims to alleviate or to improve.

2.2 Please describe the aetiology of the problem in the specific context in which your initiative is embedded. Please describe any resources (stocks) or tangible and intangible components being required or produced during the processes that cause the problem.

2.3 Please describe the processes that affect the problem in the specific context in which your initiative is embedded. Please describe relevant physical relationships as well as governance and decision making processes.

2.4 Please describe relevant stakeholders and actors who affect, are affected by, or perceive themselves to be affected by this problem and the relationships that influence it. Please focus on important societal groups, institutions or organizations. Please consider as ***stakeholders*** those individuals or institutions that affect or are affected by the problem that the initiative addresses. ***Actors*** are a subset of the stakeholders, who affect the problem that the initiative addresses. In contrast to ***team members***, they represent the interests and perspectives of societal groups and institutions rather than acting under the direction of the project or program managers of the initiative.

2.5 What are the overall objectives of the initiative? This question refers to the immediate outputs and outcomes that your initiative aspires to achieve.

2.6 How would achieving the initiatives' objectives lead to wider impacts? This question refers to the initiative's theory of change, i.e. the mechanisms by which the initiatives' outputs and outcomes translate into wider impacts beyond the initiatives' control to resolve the shortcoming or gaps that it addresses.

2.7 How do you measure the success of your initiative? This question refers to the initiative's criteria of success – how you judge that you have achieved your objectives.

2.8 What do you consider the most important benefits / accomplishments of this initiative? This question refers to accomplishments of the initiatives' process, outcomes and impacts up to date. It serves to complement or contrast with the envisioned / intended objectives. It refers both to process, expected outcomes as measured by the criteria of success, impacts as specified in the theory of change, and unintended or unexpected consequences. Please provide only observed benefits. Please do not provide planned benefits that are not completed or have not been operational long enough to provide observed benefits.

2.9 What do you consider the most important challenges / shortcomings of this initiative? This question refers to shortcomings of the initiatives' process, outcomes and impacts up to date. It serves to complement or contrast with the envisioned / intended objectives. It refers both to shortcomings of process, expected outcomes as measured by the criteria of success, impacts as specified in the theory of change, and unintended or unexpected consequences. Please provide only observed benefits. Please do not provide planned benefits that are not completed or have not been operational long enough to provide observed benefits.

3. Thinking

INCLUSIVE DESIGN PROCESS

3.1 **How are objectives and their relative importance established?** Conflicting objectives, limited resources or external constraints require implicit or explicit trade-offs that reflect the importance of objectives. Please score the attention that was/is devoted to developing and understanding objectives, trade-offs and degrees of importance.

☐ no attention ☐ implicit attention, but no explicit deliberation ☐ explicit deliberation without formal analysis ☐ formal analysis ☐ don't know / NA

3.2 **Has a theory of change been elaborated to match the objectives of the initiative?** A theory of change specifies how the initiative will produce impact that transforms the problem that it addresses. Please score the attention that was devoted to understanding and validating the changes that the initiative needs to bring about to achieve its objectives.

☐ no attention ☐ implicit attention, but no explicit deliberation ☐ explicit deliberation without formal analysis ☐ formal analysis ☐ don't know / NA

3.3 **How do the objectives and the theory of change reflect multiple perspectives, value systems and beliefs?** This question refers to the openness and inclusiveness of the process of seting-up the initiative.

☐ they reflect the perspective of the initiative's leadership
☐ they were decided by the initiative's leadership and aim at reflecting multiple perspectives, but without dialogue or negotiation with actors and stakeholders
☐ they after dialogue with actors and stakeholders, but without negotiation
☐ they were negotiated and agreed upon by the initiative's leadership, actors and stakeholders

☐ don't know / NA

3.4 How do the methods, scales and criteria of success reflect multiple perspectives, value systems and beliefs? This question refers to the openness and inclusiveness of designing the implementation phase.

☐ they reflect the perspective of the initiative's leadership
☐ they were decided by the initiative's leadership and aim at reflecting multiple perspectives, but without dialogue or negotiation with actors and stakeholders
☐ they after dialogue with actors and stakeholders, but without negotiation
☐ they were negotiated and agreed upon by the initiative's leadership, actors and stakeholders

☐ don't know / NA

CONSIDERATION OF SYSTEM CHARACTERISTICS

3.5 How many dimensions does the defined problem encompass? TO BE ANSWERED FROM TABLE 1! Not to be asked to the interviewee at this point!

☐ One ☐ Two ☐ Three to four ☐ More than four ☐ don't know / NA

3.6 Is the problem that the initiative addresses an event, a pattern, or a structure? *Events* are singular; and we can only react to events. ***Patterns*** let us understand reality at a deeper level, i.e. trends, which are changes over time to which we can adapt. Thinking at a ***structural*** level means thinking in terms of causal connections.

☐ events ☐ patterns ☐ structures ☐ don't know / NA

3.7 How are time delays between different processes that lead to the problem considered? Please score the attention that was devoted to understanding time scales and delays between different processes.

☐ no attention ☐ implicit attention, but no explicit deliberation ☐ explicit deliberation without formal analysis ☐ formal analysis ☐ don't know / NA

3.8 **How are feedback loops and causal interactions between different processes that lead to the problem considered?** Feedback loops are interactions between two or more factors that mutually reinforce (positive feedback loop), or control (negative feedback loop) each other. Please score the attention that was devoted to understanding feedback loops and causal interactions.

☐ no attention ☐ implicit attention, but no explicit deliberation ☐ explicit deliberation without formal analysis ☐ formal analysis ☐ don't know / NA

LEVERAGE POTENTIAL

3.9 **Does the initiative comprehensively translate the problem into scientific or developmental questions?** This question asks, whether the research questions and developmental questions reflect the complexity of different aspects of the problem.

☐ scientific or developmental questions do **not** address **any** aspects the problem
☐ scientific or developmental questions address **few** aspects of the problem
☐ scientific or developmental questions address **many** aspects of the problem
☐ scientific or developmental questions address **all** aspects of the problem

☐ don't know / NA

3.10 **Where is the initiative situated in relation to the chain of events causing the problem?** This question should be answered by putting into perspective the theory of change, the project impacts according to its objectives, and the problem that has been identified in the system.

☐ correcting damage ☐ preventing damage ☐ redirecting the problem ☐ removing the cause ☐ don't know / NA

3.11 Please use **column 1 to 3 of Table 1 (next pages)** to specify how the initiative's **OBJECTIVES** aim to address different **DIMENSIONS** of the problem, and which **SCALES** are suitable to measure its impact. A "dimension" groups entities that can be captured by the same scale or concept. It is up to the respondent to introduce dimensions that express the project's objectives. Start by specifying objectives and by selecting which dimensions they address. Describe the relevant dimensions for the initiative (there may be fewer or more than the lines in table 1). Consider the theory of change. What is the initiative aiming to have an impact on? And which dimensions may support or limit the outcomes and impacts of the initiative?

Examples of dimensions could be: geographical space (scales within this dimension could be e.g. local, regional, global….), time (scales e.g. hours, days, years, centuries…), dimension of life (scales: e.g. cells tissues, organs, individuals, populations, ecosystems….), network/organisation (e.g. network of researchers, individuals from the public, institutions and national ministries, laboratories...), economy (e.g. farm level, sector-wise, national, regional, international trade, benefits, costs....), legislation (e.g. bilateral agreements, national laws), governance (political dimension, e.g. international/national strategy, local research project, specific task/work package), value constructs (e.g. interest groups, NGOs, religious beliefs, international standards......), collaborative capabilities (trust, networks, mutual understanding,…), or any other.

Please use **column 4** to list the **ACTORS** and **STAKEHOLDERS** (individuals, groups, or institutions that affect, or are affected by the system that the initiative targets), ***that are involved in the initiative and relevant for achieving each objective***. Please consider representatives of different academic disciplines, societal sectors, governmental or corporate organizations, for-profit or non-profit institutions and interest groups, as well as the general public as potential stakeholders.

Please use **column 5 of Table 1 (next pages)** to score how strongly it would affect the impacts of the initiative on the addressed problem, if any particular objective was excluded? Please score on a scale from "no relevance" to "essential".

Please use **column 6 of Table 1 (next pages)** to score how strongly the problem is affected by each dimension? Please score on a scale from "no relevance" to "essential".

Please use **column 7 of Table 1 (next pages)** to score how well the importance of each dimension in the initiative matches the degree that the problem is affected by the dimension? This question aims at scoring the match between the initiative and the system within which it operates. Please consider the scores from columns five and six to assess the match and score on a scale from "no match" to "perfect match".

TABLE 1						
1) Objective	***2) Dimension***	***3) Scales***	***4) Involved stakeholder***	***5) Effect of excluding objective***	***6) Effect of dimension on problem***	***7) Initiative-context match***
				☐ no relevance<br>☐ some<br>☐ highly<br>☐ essential<br><br>☐ don't know	☐ no relevance<br>☐ some<br>☐ highly<br>☐ essential<br><br>☐ don't know	☐ no match<br>☐ weak<br>☐ strong<br>☐ perfect<br><br>☐ don't know
				☐ no relevance<br>☐ some<br>☐ highly<br>☐ essential<br><br>☐ don't know	☐ no relevance<br>☐ some<br>☐ highly<br>☐ essential<br><br>☐ don't know	☐ no match<br>☐ weak<br>☐ strong<br>☐ perfect<br><br>☐ don't know
				☐ no relevance<br>☐ some<br>☐ highly<br>☐ essential<br><br>☐ don't know	☐ no relevance<br>☐ some<br>☐ highly<br>☐ essential<br><br>☐ don't know	☐ no match<br>☐ weak<br>☐ strong<br>☐ perfect<br><br>☐ don't know
				☐ no relevance<br>☐ some<br>☐ highly<br>☐ essential<br><br>☐ don't know	☐ no relevance<br>☐ some<br>☐ highly<br>☐ essential<br><br>☐ don't know	☐ no match<br>☐ weak<br>☐ strong<br>☐ perfect<br><br>☐ don't know
				☐ no relevance<br>☐ some<br>☐ highly<br>☐ essential<br><br>☐ don't know	☐ no relevance<br>☐ some<br>☐ highly<br>☐ essential<br><br>☐ don't know	☐ no match<br>☐ weak<br>☐ strong<br>☐ perfect<br><br>☐ don't know
				☐ no relevance<br>☐ some<br>☐ highly<br>☐ essential<br><br>☐ don't know	☐ no relevance<br>☐ some<br>☐ highly<br>☐ essential<br><br>☐ don't know	☐ no match<br>☐ weak<br>☐ strong<br>☐ perfect<br><br>☐ don't know

TABLE 1 (continued)						
1) Objective	***2) Dimension***	***3) Scales***	***4) Involved stakeholder***	***5) Effect of excluding objective***	***6) Effect of dimension on problem***	***7) Initiative-context match***
				☐ no relevance<br>☐ some<br>☐ highly<br>☐ essential<br><br>☐ don't know	☐ no relevance<br>☐ some<br>☐ highly<br>☐ essential<br><br>☐ don't know	☐ no match<br>☐ weak<br>☐ strong<br>☐ perfect<br><br>☐ don't know
				☐ no relevance<br>☐ some<br>☐ highly<br>☐ essential<br><br>☐ don't know	☐ no relevance<br>☐ some<br>☐ highly<br>☐ essential<br><br>☐ don't know	☐ no match<br>☐ weak<br>☐ strong<br>☐ perfect<br><br>☐ don't know
				☐ no relevance<br>☐ some<br>☐ highly<br>☐ essential<br><br>☐ don't know	☐ no relevance<br>☐ some<br>☐ highly<br>☐ essential<br><br>☐ don't know	☐ no match<br>☐ weak<br>☐ strong<br>☐ perfect<br><br>☐ don't know
				☐ no relevance<br>☐ some<br>☐ highly<br>☐ essential<br><br>☐ don't know	☐ no relevance<br>☐ some<br>☐ highly<br>☐ essential<br><br>☐ don't know	☐ no match<br>☐ weak<br>☐ strong<br>☐ perfect<br><br>☐ don't know
				☐ no relevance<br>☐ some<br>☐ highly<br>☐ essential<br><br>☐ don't know	☐ no relevance<br>☐ some<br>☐ highly<br>☐ essential<br><br>☐ don't know	☐ no match<br>☐ weak<br>☐ strong<br>☐ perfect<br><br>☐ don't know
				☐ no relevance<br>☐ some<br>☐ highly<br>☐ essential<br><br>☐ don't know	☐ no relevance<br>☐ some<br>☐ highly<br>☐ essential<br><br>☐ don't know	☐ no match<br>☐ weak<br>☐ strong<br>☐ perfect<br><br>☐ don't know

1. Planning

IDENTIFICATION AND ENGAGEMENT OF SECTORS, ACTORS AND STAKEHOLDERS

4.1 **How are sectors, disciplines, stakeholders and actors identified, that affect or are affected by the problem that the initiative targets and are thus relevant for achieving its objectives and for leveraging impact?** Sector refer to societal or institutional groups that share common aims or similar institutional structures, such as banking, industry, or public governance authorities. Disciplines refer to scientific specializations such as medicine (and veterinary medicine), mathematics, ecology, chemistry, environmental science. Actors and stakeholders are individuals, groups or institutions, who have a vested interest, or dispose of knowledge which is relevant for achieving the initiative's objectives. Actors additionally affect the problem and are thus crucial for leveraging impact.

☐ no identification of relevant sectors, disciplines, actors and stakeholders
☐ ad hoc identification of some sectors, disciplines, stakeholders and actors
☐ informal process, aimed at comprehensive selection of relevant sectors, disciplines, actors and stakeholders
☐ formal analysis, consultations with external experts/advisors or participatory process

☐ don't know / NA

4.2 **How is actor and stakeholder commitment assured?** This question refers to the degree of formality and institutional commitment invested in / required from stakeholder groups.

☐ no stakeholder engagement
☐ ad hoc invitations according to immediate opportunity
☐ engagement according to informal agreements
☐ formal engagement negotiated with stakeholders and institutional commitments to ensure accountability

☐ don't know / NA

REFLEXIVITY AND ADAPTIVENESS

4.3 **Which opportunities for reflection and self-assessment does the initiative provide?** This question refers to the effort and opportunities within the initiative to enable reflexivity among team members and stakeholders. It can refer to both formal arrangements or other occasions, in which project management, team members and stakeholders of the initiative ask for, and receive feedback on process, accomplishments and future directions.

☐ no opportunities for self-assessment and reflection
☐ informal ad hoc opportunities for internal dialogue, feedback and reflection
☐ structured process for internal dialogue, feedback and reflection
☐ structured process for internal dialogue and feedback that requires team members and stakeholders to personally experience and take perspectives that are different from one's own (e.g. temporary role changes, joint field work etc.)

☐ don't know / NA

4.4 **How flexible is the initiative's execution and timeline to respond to internal or external changes in the short-, mid-, and long term?** This question refers to opportunities within the initiative to adapt to changing external (e.g. environmental situation) or internal conditions (e.g. policy, funding, or management structures). It can both refer to institutional arrangements for future adaptations, or past experience.

	**short-term**<br><= 1 year	**mid-term**<br>1-3 years	**long-term**<br>>= 3 years
no possibility to adapt the original plan during its execution	☐	☐	☐
minor adjustments possible according to circumstances	☐	☐	☐
considerable possibility to adjust according to circumstances	☐	☐	☐
formal iterative decision making and management process to regularly reconsider and adapt execution	☐	☐	☐
don't know / NA	☐	☐	☐

COMPETENCES & METHODS

4.5 **How adequate are the competences of team members and actors to achieve the objectives?** There is no generalizable scale for the adequacy of competences across different contexts, and this question can easily lead to strategically biased responses. Therefore, the scale is up to the evaluator's professional judgement, which shall take into account local context and perspectives, and draw on a broad data basis.

☐ inadequate ☐ partly adequate ☐ mostly adequate ☐ entirely adequate ☐ don't know / NA

4.6 **How adequate are the methods to achieve the objectives?** There is no generalizable scale for the adequacy of methods across different contexts, and this question can easily lead to strategically biased responses. Therefore, the scale is up to the evaluator's professional judgement, which shall take into account local context and perspectives, and draw on a broad data basis.

☐ inadequate ☐ partly adequate ☐ mostly adequate ☐ entirely adequate ☐ don't know / NA

RESOURCE ALLOCATION

4.7 **How adequate are the budget allowances to achieve the objectives?** There is no generalizable scale for the adequacy of the allowances across different contexts, and this question can easily lead to strategically biased responses. Therefore, the scale is up to the evaluator's professional judgement, which shall take into account local context and perspectives, and draw on a broad data basis.

☐ inadequate ☐ partly adequate ☐ mostly adequate ☐ entirely adequate ☐ don't know / NA

4.8 How adequate are the time allowances to achieve the objectives? There is no generalizable scale for the adequacy of allowances across different contexts, and this question can easily lead to strategically biased responses. Therefore, the scale is up to the evaluator's professional judgement, which shall take into account local context and perspectives, and draw on a broad data basis.

☐ inadequate ☐ partly adequate ☐ mostly adequate ☐ entirely adequate ☐ don't know / NA

2. Organization

INTERNAL TEAM STRUCTURE

Please consider as team members those individuals, who participate in the initiative under the direction of the project or program management and contribute specific skills or fulfil specific duties to achieve the initiative's objectives. In contrast to stakeholders, they do not primarily participate in the initiative as representatives of interests and perspectives of societal groups or organizations.

5.1 If more than one team are present, how are inter-team relations? This question refers to the collaborative spirit between different teams. Since this question is prone to produce biased responses, it is therefore crucial to validate responses with various participants at different hierarchical levels. If relation between teams differ, please score an average value.

☐ competition ☐ ignorance ☐ mutual information ☐ mutual support ☐ don't know / NA

5.2 How are the team objectives established? This question explores the degree of formality employed in assigning team objectives.

☐ not at all ☐ implicitly assumed ☐ ad hoc agreed ☐ explicitly defined ☐ don't know / NA

5.3 How are individual roles established and differentiated? This question explores the degree of formality employed in assigning team member's roles and responsibilities.

☐ not at all ☐ implicitly assumed ☐ ad hoc agreed ☐ explicitly defined ☐ don't know / NA

EXTERNAL ACTOR AND STAKEHOLDER NETWORK

5.4 **How frequently are actors and stakeholders involved in the initiative?** This question refers to the frequency of any kind of interaction and information exchange between actors, stakeholders and team members of the initiative. In case the frequency differs between different stakeholders or actor groups, please score the average frequency of interaction with each core actor or stakeholder.

☐ never ☐ occasionally, but not on a regular basis ☐ in regular intervals but not frequently ☐ frequently ☐ don't know / NA

5.5 **How intense is the collaboration with actors and stakeholders in the initiative?** In case the intensity differs between different actors or stakeholder groups, please score the average intensity of collaboration with core actors and stakeholders.

☐ No contact or indirect information exchange through the initiative, but no face-to face participation
☐ Face-to face participation in events, but no joint task execution
☐ Joint task execution, but without an influence on decision making or initiative steering
☐ Joint steering, decision making, or co-leadership

☐ don't know / NA

BRIDGING KNOWLEDGES

5.6 **Which <u>methods</u> are used to 'bridge', 'link' or 'integrate' the knowledge of team members, actors and stakeholders?** Since terminology and methodology of knowledge integration are not commonly shared between different initiatives, considerable depth of inquiry and effort to translate interview responses into answers to this question are required from the evaluator. Answers to this question draw on the following sources:

- Scholz et al. 2002: Embedded case study methods: integrating quantitative and qualitative knowledge. Sage Publications.
- Bergmann et al. 2012: Methods for transdisciplinary research: a primer for practice. Frankfurt, New York: Campus.
- Hoffmann et al. 2017: Methods and procedures of transdisciplinary knowledge integration. Ecology and Society 22/1.

If appropriate, please tick multiple elements.

☐ none
☐ written information exchange (e.g. reports dissemination, social media networks, mail)
☐ face-to face networking & unstructured dialogue (e.g. receptions, information events)
☐ facilitated dialogue/moderation (e.g. workshops, focus groups, panel discussions)
☐ mediation through trusted bridge persons or boundary institutions
☐ joint definition of concepts and analytical frameworks
☐ joint formulation of objectives, research questions and hypotheses
☐ joint elaboration of theory of change, methods and assessment procedures
☐ joint conduction of fieldwork or joint elaboration of products (e.g. boundary objects, models, system dynamic graphs)
☐ changes of perspective / experiential encounters

☐ other, please specify:
☐ don't know / NA

5.7 **Which processes are used to 'bridge', 'link' or 'integrate' the knowledge of team members, actors and stakeholders?** Since terminology and methodology of knowledge integration are not commonly shared between different initiatives, considerable depth of inquiry and effort to translate interview responses into answers to this question are required from the evaluator. Answers to this question draw on the following sources:

- Rossini 1979: Frameworks for integrating interdisciplinary research. Research Policy 8/1.
- Hoffmann et al. 2017: Methods and procedures of transdisciplinary knowledge integration. Ecology and Society 22/1.

If appropriate, please tick multiple elements.

☐ none
☐ centralized integration through leader / project management
☐ integration through negotiation among experts
☐ common group learning

☐ other, please specify:
☐ don't know / NA

3. Working

POWER DISTRIBUTION

6.1 **How is the distribution of power or influence between team members and stakeholders from different…** Please consider formal power (e.g. positions, hierarchies, budget responsibilities) as well as informal or "soft" forms of influence (e.g. openness to voice opinions, respect towards expertise and personality of team members, loyalties between team members).

**Disciplines or sectors?**	☐ concentrated	☐ unbalanced	☐ mostly balanced	☐ balanced	☐ don't know / NA
**Social classes or gender?**	☐ concentrated	☐ unbalanced	☐ mostly balanced	☐ balanced	☐ don't know / NA
**Ethnicities, cultures or religions?**	☐ concentrated	☐ unbalanced	☐ mostly balanced	☐ balanced	☐ don't know / NA

LEADERSHIP

6.2 **Is the management structure adequate to support the team and actors in achieving the initiative's objectives?** This question refers to the ability of the project management to coordinate and administer initiative activities. Due to the lack of a generalizable scale to aggregate the various dimensions related to project coordination, this question relies on the evaluators judgement, and requires drawing on input from multiple initiative participants.

☐ inadequate ☐ partly adequate ☐ mostly adequate ☐ entirely adequate ☐ don't know / NA

6.3 How would you characterize the leadership approach to project management? This question refers to definitions by Yukl (*Effective Leadership Behavior: What We Know and What Questions Need More Attention. Acad. of Manag. Perspect. 26/4: 66–85 (2012)*). It requires considerable inquiry on behalf of the evaluator. ***Task-oriented*** leadership focuses on accomplishing work in an efficient and reliable way. ***Relations-oriented*** leadership aims at increasing the quality of human resources and relations ("human capital"). ***Change-oriented*** leadership focuses on increased innovation, collective learning, and adaptation to the external environment. If appropriate, please tick multiple elements.

☐ no leadership ☐ task-oriented ☐ relationship-oriented ☐ change-oriented ☐ don't know / NA

6.4 How open-minded is the leadership to creative input? This question refers to the openness of the initiatives leadership to consider and test creative or unconventional ideas and input. To assess this question, it is suggested to ask participants for information and suggestions that were creative in content or did not follow conventional chains of command, and whether any of these led to surprising decisions, unconventional approaches differing from previous habits or protocols, or unexpected changes in project design, fieldwork and objectives.

☐ closed ☐ rarely open ☐ usually open ☐ frequently open-minded ☐ don't know / NA

6.5 How flexible are internal hierarchies and decision making in adapting to circumstances and tasks? The question refers to the degree of independence participants are given according to their expertise and skills, and the leaderships´ flexibility to devolve decision making according to need and context. To assess this question, it is suggested to ask for the degree of independence participants are given in specific realms, and for occasions, in which unconventional situations required deviating from chains of command in unforeseen ways.

☐ hierarchies are formalistic, top-down decision making
☐ hierarchies are formalistic, top-down decision making, but takes lower level's perspectives into account
☐ hierarchies are formalistic, but decisions reflect cross-hierarchical perspectives
☐ hierarchies are flexible and reflect the expertise and experience needed in specific situations

☐ don't know / NA

CONFLICT RESOLUTION

6.6 **How does the leadership manage tensions and conflicts?** This question addresses the degree to which project management involves conflicting partners to solve conflicts in the initiative. 'Evasion' designates a leadership that does not address tensions or conflicts within the initiative. 'Appeasement' is based on satisfying demands brought forward in a conflict without involving all parties. 'Imposition' prescribes certain solutions to conflicting parties. 'Mediation' negotiates solutions among conflicting parties. If appropriate, please tick multiple elements.

☐ evasion ☐ appeasement ☐ imposition ☐ mediation ☐ don't know / NA

6.7 **At what level are conflicts resolved?** This question addresses the degree of reflection and learning that project management derives from conflicts within the initiative. 'Hierarchical' refers to solutions that are prescribed without reflecting on sources and potential lessons. Reflection and learning can address mistakes and potential changes to 'factual' causes, can search for and address 'emotional' sources of conflict in individual personalities, or serve to build coherence, understanding, trust, and commitment across the entire team. If appropriate, please tick multiple elements.

☐ hierarchical ☐ factual ☐ emotional & personal growth ☐ team building ☐ don't know / NA

6.8 **How does the team react to conflict?** This question refers to conflict-related attitudes and group dynamics in the entire initiative. Conflicts and sources of potential future conflicts such as unwelcome information can be seen as negative and to be avoided, thus leading to silencing or 'concealing'. 'Confrontation' spells conflict out, while focusing on the conflictual situation and a resistance to reflect own positions. 'Dialogue' spells conflict out, but with an attitude focused on solutions and reflecting own positions. Tolerant and resilient conflict behaviour addresses conflicts in dialogue, but also accepts differences in objectives and perceptions, acknowledges their justification and accommodates certain levels of ambiguity and diversity in interpreting conflictive situations. If appropriate, please tick multiple elements.

☐ concealing ☐ confrontation ☐ dialogue ☐ tolerance & resilience ☐ don't know / NA

4. Sharing

PROCESSES FOR INFORMATION EXCHANGE

7.1 How adequate are the resources allocated to ensure information sharing? This refers to both monetary and non-monetary resources required for enabling information sharing.

☐ inadequate ☐ partly adequate ☐ mostly adequate ☐ entirely adequate ☐ don't know / NA

7.2 How are agreements concerning information sharing established?

☐ not at all ☐ implicit agreement, without explicit negotiation ☐ explicit negotiation, but without a binding document ☐ binding document ☐ don't know / NA

7.3 Does the initiative have processes to facilitate exchange of information within the initiative and are these used? E.g. newsletters, workshops, reports, publications, online sharing platform. Please only consider processes which are also used.

☐ none ☐ few and irregularly ☐ several or regularly ☐ several and regularly ☐ don't know / NA

7.4 Does the initiative have processes to facilitate exchange of information beyond the initiative and are these used? E.g. newsletters, workshops, reports, publications, online sharing platform. Please only consider processes which are also used.

☐ none ☐ few and irregularly ☐ several or regularly ☐ several and regularly ☐ don't know / NA

DATA SHARING

7.5 **How adequate are the procedures to ensure the quality of shared data?** E.g. completeness, error-checking, clear and accurate descriptions of variables and of calculations, available documentation.

☐ inadequate ☐ partly adequate ☐ mostly adequate ☐ entirely adequate ☐ don't know / NA

7.6 **How adequate are the procedures to ensure safe and appropriate data storage and accessibility?** E.g. is the storage safe and protected, is extraction of data feasible without access to data managers, or are expert managers readily available for extraction of data, is the process of data extraction bureaucratic/cumbersome/overly time-consuming.

☐ inadequate ☐ partly adequate ☐ mostly adequate ☐ entirely adequate ☐ don't know / NA

7.7 **How well / how much are data being shared within the initiative?**

☐ not at all ☐ between few people ☐ between few groups ☐ within entire initiative ☐ don't know / NA

METHODS AND RESULTS SHARING

7.8 **How well / how much are methods shared within the initiative?**

☐ not at all ☐ between few people ☐ between few groups ☐ within entire initiative ☐ don't know / NA

7.9 **How well / how much are results shared within the initiative?**

☐ not at all ☐ between few people ☐ between few groups ☐ within entire initiative ☐ don't know / NA

INSTITUTIONAL MEMORY

7.10 Does the initiative create long-term institutional knowledge reservoirs for data, methods and results? E.g. publications, detailed reports/manuals, database descriptions, standard operating procedures, introductions to inform new staff about essential procedures.

☐ not at all ☐ few ☐ several ☐ comprehensively ☐ don't know / NA

7.11 How adequate are the procedures to safe-guard access to data, information and results in case of system change or external disturbances? E.g. change of IT-system, data ownership, institutional organization, software breakdowns, or loss of key personnel.

☐ inadequate ☐ partly adequate ☐ mostly adequate ☐ entirely adequate ☐ don't know / NA

5. Learning

INDIVIDUAL LEARNING

8.1 How often do individuals receive information which may be understood and may potentially lead to learning, but it is not put into practice in or outside the initiative by the individuals (basic learning)?

☐ never ☐ rarely ☐ regularly ☐ frequently ☐ don't know / NA

8.2 How often is information understood, learnt and applied to improve procedures, competencies, technologies and paradigms without challenging the individuals' underlying beliefs and assumptions (adaptive learning)?

☐ never ☐ rarely ☐ regularly ☐ frequently ☐ don't know / NA

8.3 How often is information understood and learnt by individuals and applied to improve procedures, competencies, technologies and paradigms as a result of modified underlying beliefs and norms of individuals (generative learning)?

☐ never ☐ rarely ☐ regularly ☐ frequently ☐ don't know / NA

TEAM LEARNING

8.4 How often do teams meet to exchange information for reporting purposes (basic learning)?

☐ never ☐ rarely ☐ regularly ☐ frequently ☐ don't know / NA

8.5 When teams meet, how often are different views presented, defended and discussed to find the best view to support decision making (adaptive learning)?

☐ never ☐ rarely ☐ regularly ☐ frequently ☐ don't know / NA

8.6 When teams meet, how often are complex issues explored through dissection of views and assumptions of team members resulting in a move towards building new ideas, views or approaches (generative learning?

☐ never ☐ rarely ☐ regularly ☐ frequently ☐ don't know / NA

ORGANIZATIONAL LEARNING

8.7 How often is existing/circulating information and knowledge collected and stored (basic learning)?

☐ never ☐ rarely ☐ regularly ☐ frequently ☐ don't know / NA

8.8 How often is collected information shared, discussed and acted upon at various levels within the organisation(s) (adaptive learning)?

☐ never ☐ rarely ☐ regularly ☐ frequently ☐ don't know / NA

8.9 How often is collected information shared, discussed and leads to change in fundamentals and objectives across all levels within the organisation(s) (generative learning)?

☐ never ☐ rarely ☐ regularly ☐ frequently ☐ don't know / NA

DIRECT ENVIRONMENT

8.10 How often is the direct environment of the initiative (involved stakeholders) **supportive for adaptive learning?** To score this question please consider in how far the direct environment accepts learning that focuses on correcting or improving existing procedures, processes, competences and technologies, as compared to expecting from you to never change procedures and processes and to never make mistakes.

☐ never ☐ rarely ☐ regularly ☐ frequently ☐ don't know / NA

8.11 How often is the direct environment of the initiative (involved stakeholders) **supportive for generative learning?** To score this question please consider in how far the general environment accepts learning that focuses on questioning the existing norms and that encourages looking beyond the existing situation, as compared to expecting from you to always adhere to existing norms and established paradigms.

☐ never ☐ rarely ☐ regularly ☐ frequently ☐ don't know / NA

GENERAL ENVIRONMENT

8.12 How often is the general environment (e.g. culture, economics, political situation) **of the initiative supportive for adaptive learning**? To score this question please consider in how far the general environment accepts learning that focuses on correcting or improving existing procedures, processes, competences and technologies, as compared to expecting from you to never change procedures and processes and to never make mistakes.

☐ never ☐ rarely ☐ regularly ☐ frequently ☐ don't know / NA

8.13 How often is the general environment (e.g. culture, economics, political situation) **of the initiative supportive for generative learning?** To score this question please consider in how far the general environment accepts learning that focuses on questioning the existing norms and that encourages looking beyond the existing situation, as compared to expecting from you to always adhere to existing norms and established paradigms.

☐ never ☐ rarely ☐ regularly ☐ frequently ☐ don't know / NA

6. Final considerations

9.1 Do you have any immediate feedback for us?

Thank you for your time and patience!!!
