# Supplementary material for: One Health evaluation of brucellosis control in Kazakhstan
Source: PLoS One. 2022 Nov 2;17(11):e0277118. doi: 10.1371/journal.pone.0277118 (PMC9629608; doi:10.1371/journal.pone.0277118)
Supplement: S3 File — (PDF) [file pone.0277118.s003.pdf]

S3. Semi-structured interview guide

Instructions to Interviewer: Use approved interview questionnaire and fill it out during the interview either manually or digitally. Assign scores. Establish rapport throughout the interview with prompts if necessary

Welcome and Introduction

Thanks for taking the time to talk with me today and providing verbal consent. In this interview, I am going to ask you a few questions relating to your experiences with the brucellosis control program. There are number of areas/domains we want to ask questions about. There are no right or wrong answers. The interview should take approximately 120-150 minutes and with your permission it will be digitally-recorded to ensure that all key points are accurately documented. Any identifying information that you use in the course of our discussion will be removed from the interview transcripts. If you wish to end the interview before I have asked all of the questions or if you wish to withdraw from the study you are free to do so.

After the NEOH questionnaire, ask the participants the two questions on acquisition of the systems thinking.

Closing remarks

At the end of the interview thank the respondent for their time, patience, and contribution. Ask if they have immediate feedback/comments for you.

Interview Domains

Domain 1. Personal information and role of the respondent

- Please state your name and title
- What is the title or name of the project or initiative that you are working in?
- Where does this initiative take place (country, district, city)?
- What is your role in this initiative?
- What would you consider your key contribution to this initiative?

Domain 2. Definition of context and initiative

- What is the problem / information gap that your initiative addresses / tries to fill?
- Please describe the aetiology of the problem in the specific context in which your initiative is embedded.
- Please describe the processes that affect the problem in the specific context in which your initiative is embedded.
- Please describe relevant stakeholders and actors who affect, are affected by, or perceive themselves to be affected by this problem and the relationships that influence it.
- What are the overall objectives of the initiative?
- How would achieving the initiatives’ objectives lead to wider impacts?
- How do you measure the success of your initiative?
- What do you consider the most important benefits / accomplishments of this initiative?
- What do you consider the most important challenges / shortcomings of this initiative?

Domain 3. Thinking

INCLUSIVE DESIGN PROCESS

- How are objectives and their relative importance established?
- Has a theory of change been elaborated to match the objectives of the initiative?
- How do the objectives and the theory of change reflect multiple perspectives, value systems and beliefs?
- How do the methods, scales and criteria of success reflect multiple perspectives, value systems and beliefs?

CONSIDERATION OF SYSTEM CHARACTERISTICS

- Is the problem that the initiative addresses an event, a pattern, or a structure?
- How are time delays between different processes that lead to the problem considered?
- How are feedback loops and causal interactions between different processes that lead to the problem considered?

LEVERAGE POTENTIAL

- Does the initiative comprehensively translate the problem into scientific or developmental questions?
- Where is the initiative situated in relation to the chain of events causing the problem?
- How many dimensions does the defined problem encompass?

Please use **column 1 to 3 of Table 1 (next pages)** to specify how the initiative's **OBJECTIVES** aim to address different **DIMENSIONS** of the problem, and which **SCALES** are suitable to measure its impact. A "dimension" groups entities that can be captured by the same scale or concept. It is up to the respondent to introduce dimensions that express the project's objectives. Start by specifying objectives and by selecting which dimensions they address. Describe the relevant dimensions for the initiative **(there may be fewer or more than the lines in table 1).** Consider the theory of change. What is the initiative aiming to have an impact on? And which dimensions may support or limit the outcomes and impacts of the initiative?

Domain 4. Planning

IDENTIFICATION AND ENGAGEMENT OF SECTORS, ACTORS AND STAKEHOLDERS

- How are sectors, disciplines, stakeholders and actors identified, that affect or are affected by the problem that the initiative targets and are thus relevant for achieving its objectives and for leveraging impact?
- How is actor and stakeholder commitment assured?

REFLEXIVITY AND ADAPTIVENESS

- Which opportunities for reflection and self-assessment does the initiative provide?

- How flexible is the initiative's execution and timeline to respond to internal or external changes in the short-, mid-, and long term?

COMPETENCES & METHODS

- How adequate are the competences of team members and actors to achieve the objectives?
- How adequate are the methods to achieve the objectives?

RESOURCE ALLOCATION

- How adequate are the budget allowances to achieve the objectives?
- How adequate are the time allowances to achieve the objectives?

Domain 5. Internal team structure

- How many teams are present in the initiative?
- If more than one team are present, how are inter-team relations?
- How are the team objectives established?
- How are individual roles established and differentiated?

EXTERNAL ACTOR AND STAKEHOLDER NETWORK

- How frequently are actors and stakeholders involved in the initiative?
- How intense is the collaboration with actors and stakeholders in the initiative?

BRIDGING KNOWLEDGES

- Which methods are used to 'bridge', 'link' or 'integrate' the knowledge of team members, actors and stakeholders?
- Which processes are used to 'bridge', 'link' or 'integrate' the knowledge of team members, actors and stakeholders?

Domain 6. Working

POWER DISTRIBUTION

- **How is the distribution of power or influence between team members and stakeholders from different disciplines or sectors? Social classes or gender? Ethnicities, cultures or religions?**

LEADERSHIP

- Is the management structure adequate to support the team and actors in achieving the initiative's objectives?
- How would you characterize the leadership approach to project management?
- How open-minded is the leadership to creative input?

- How flexible are internal hierarchies and decision making in adapting to circumstances and tasks?

CONFLICT RESOLUTION

- How does the leadership manage tensions and conflicts?
- At what level are conflicts resolved?
- How does the team react to conflict?
-

Domain 7. Sharing

PROCESSES FOR INFORMATION EXCHANGE

- How adequate are the resources allocated to ensure information sharing?
- Does the initiative have processes to facilitate exchange of information within the initiative and are these used?
- Does the initiative have processes to facilitate exchange of information beyond the initiative and are these used?

DATA SHARING

- How adequate are the procedures to ensure the quality of shared data?.
- How adequate are the procedures to ensure safe and appropriate data storage and accessibility?
- How well / how much are data being shared within the initiative?

METHODS AND RESULTS SHARING

- How well / how much are methods shared within the initiative?
- How well / how much are results shared within the initiative?

INSTITUTIONAL MEMORY

- Does the initiative create long-term institutional knowledge reservoirs for data, methods and results?
- How adequate are the procedures to safe-guard access to data, information and results in case of system change?

Domain 8. Learning

INDIVIDUAL LEARNING

- How often do individuals receive information which may be understood and may potentially lead to learning, but it is not put into practice in or outside the initiative by the individuals (basic learning)?

- How often is information understood, learnt and applied to improve procedures, competencies, technologies and paradigms without challenging the individuals' underlying beliefs and assumptions (adaptive learning)?
- How often is information understood and learnt by individuals and applied to improve procedures, competencies, technologies and paradigms as a result of modified underlying beliefs and norms of individuals (generative learning)?

TEAM LEARNING

- How often do teams meet to exchange information for reporting purposes (basic learning)?
- When teams meet, how often are different views presented, defended and discussed to find the best view to support decision making (adaptive learning)?
- When teams meet, how often are complex issues explored through dissection of views and assumptions of team members resulting in a move towards building new ideas, views or approaches (generative learning)?

ORGANIZATIONAL LEARNING

- How often is existing/circulating information and knowledge collected and stored (basic learning)?
- How often is collected information shared, discussed and acted upon at various levels within the organisation(s) (adaptive learning)?
- How often is collected information shared, discussed and leads to change in fundamentals and objectives across all levels within the organisation(s) (generative learning)?

DIRECT ENVIRONMENT

- How often is the direct environment of the initiative (involved stakeholders) supportive for adaptive learning?
- How often is the direct environment of the initiative (involved stakeholders) supportive for generative learning?

GENERAL ENVIRONMENT

- How often is the general environment (e.g. culture, economics, political situation) of the initiative supportive for adaptive learning?
- How often is the general environment (e.g. culture, economics, political situation) of the initiative supportive for generative learning?

Acquisition of systems thinking

- Is there any specific obstacle you encountered during introduction to the systems approach?
- In retrospection, how would you have found it easiest to learn systems theory?
