# Supplementary material for: One Health evaluation of brucellosis control in Kazakhstan
Source: PLoS One. 2022 Nov 2;17(11):e0277118. doi: 10.1371/journal.pone.0277118 (PMC9629608; doi:10.1371/journal.pone.0277118)
Supplement: S6 File — (PDF) [file pone.0277118.s006.pdf]

EVOLvINC	scores	NEOH	scores
**Thinking**	0.33	**Thinking**	0.4
Inclusive design process	0.33	Dimensions coverage + balance	0.6
Systems characteristics	0.33	Initiative-to-environment match	0.8
Leverage potential	0.83	Integrated health approach	0.8
		System features and targets	0.3
		Sustainability and socio-ecological considerations	0.5
		Perspectives and TOC-factors	0.3
**Planning**	0.58	**Planning**	0.73
Stakeholder engagement	0.83	Common aims	0.6
Reflexivity and adaptiveness	0.50	Stakeholder and actor engagement	0.9
Competences & methods	0.66	Self-assessment and plan revisions	0.9
Resource allocation	0.50	Objective 1 - Annual surveillance - prevalence	0.8
		Objective 2 - Timely slaughter of positive animals	0.6
		Objective 3 - Human incidence	0.8
**Working**	0.80	**Working**	0.7
Power distribution	1.00	Broadness of initiative	0.6
Leadership	0.66	Collaboration	0.8
Conflict resolution	0.66	Transdisciplinary balance	0.7
		Cultural and social balance	1
		Flexibility and adaptation	0.4
**Sharing**	0.74	**Sharing**	0.66
Processes for information exchange	0.66	General information/awareness sharing	0.7
Data	0.66	Data and information sharing	0.8
Methods and results	0.83	Methods and results sharing	0.8
Institutional memory	0.83	Institutional memory/resilience	0.8
**Learning**	0.66	**Learning**	0.51
Individual learning	0.33	Focus on adaptive and generative individual learning	0.4
Team learning	0.66	Focus on adaptive and generative team learning	0.5
Organizational learning	0.66	Focus on adaptive and generative organisational learning	0.5
Direct environment	0.66	Direct learning environment supportive of adaptive and generative learning	0.8
General environment	0.67	General learning environment supportive of adaptive and generative learning	0.5
**Organisation**	0.83	**Systemic organisation**	0.66
Internal team structure	1.00	Team structures	0.9
External actor and stakeholder network	0.83	Social and leadership structures + skills	0.528
Bridging knowledges	0.17	Competence	0.7
		Focus and innovation	0.6
